# Perceptual metacognition and self-esteem: the role of feedback valence in local and global monitoring bias

**DOI:** 10.1186/s40359-025-03089-x

**Published:** 2025-09-22

**Authors:** Guanzhuo Zhang, Jiafeng Zhang, Hui Li, Wei Liu, Xuemin Zhang

**Affiliations:** 1 Beijing Key Laboratory of Applied Experimental Psychology, National Demonstration Center for Experimental Psychology Education, Faculty of Psychology, Beijing Normal University, No. 19, Xinjiekouwai Avenue, Beijing, 100875 China; 2 State Key Laboratory of Cognitive Neuroscience and Learning and IDG, McGovern Institute for Brain Research, Beijing Normal University, Beijing, China

**Keywords:** Perceptual metacognition, Metacognitive monitoring bias, Low self-esteem, Local metacognition, Global meatcognition

## Abstract

Previous studies suggest that individuals with low self-esteem tend to undervalue themselves, a tendency that may reflect a metacognitive monitoring bias, which in turn can be shaped by feedback valence. However, research specifically exploring how self-esteem and feedback valence affect metacognitive monitoring remains scarce. Through three experiments, the study revealed that individuals tend to exhibit an underestimation bias in perceptual metacognitive monitoring. However, compared to individuals with high self-esteem, those with low self-esteem exhibited more pronounced local and global monitoring biases in the absence of feedback. Furthermore, feedback valence influenced both local and global metacognitive monitoring in low self-esteem individuals, whereas its effects in high self-esteem individuals were limited to global monitoring. Additionally, feedback valence reduced global monitoring disparities between self-esteem groups, while only positive feedback narrowed the gap in local monitoring. These findings suggest that the influence of feedback valence on metacognitive monitoring biases varies depending on both self-esteem level and the level of monitoring. These results provide valuable insights into the interplay among self-esteem, metacognitive monitoring and feedback, with implications for the development of targeted educational strategies, particularly for individuals with low self-esteem.

**Supplementary Information:**

The online version contains supplementary material available at 10.1186/s40359-025-03089-x.

## Introduction

Metacognitive monitoring, a fundamental aspect of metacognition, involves the real-time observation and evaluation of one’s cognitive processes during task execution [4, 22]. Accurate metacognitive monitoring is essential for enhancing learning, decision-making, problem-solving, self-regulation, and self-control [1, 42, 53]. However, inaccuracies in metacognitive monitoring are common, often manifesting as overestimation or underestimation of task performance compared to objective outcomes, a phenomenon known as monitoring bias [11, 31, 33]. An increasing body of research supports the view that the metacognitive monitoring should be delineated into two distinct dimensions: local and global level [8, 35, 38, 50]. Local monitoring refers to participants’ appraisal of their performance at each stage during the overall task [8, 16, 23, 35], whereas global monitoring involves an overall rating of their performance after completing the task [38, 47]. In laboratory research, participants are usually required to complete a task with many trials, such as a two-alternative forced choice (2-AFC) task. The local monitoring bias is usually calculated by the mean difference between their object performance and their subjective confidence in each trial. In contrast, the global monitoring bias is measured by the difference between the overall accuracy and the overall rating of their performance after participants finished the task.

Numerous researchers have investigated how monitoring biases manifest at both levels. For example, in the domain of knowledge-based decision-making, individuals tend to overestimate their performance at the local level, as indicated by the mean judged confidence exceeds overall percentage of correct answers [19, 35, 37]. In contrast, at the global level, individuals typically underestimate their performance, as evidenced by global confidence ratings falling below their actual accuracy [21, 35, 54]. In the memory domain, healthy individuals tend to underestimate their performance not only at the global level [20, 24] but also at the local level [24]. These results suggested that the directional tendencies are not always consistent in global and local monitoring biases. However, these studies represent the high-order cognitive process, reflecting a more complex mechanism. In order to figure out the underlying mechanism of the monitoring bias, seeking breakthrough points from the fundermental cognitive processes (shcu as perception, [25]) may be a better way.

Previous studies on perceptual metacognition primarily focused on whether individuals can distinguish the clarity of stimuli in peripheral vision [9, 25, 55]. For example, a study examined local and global monitoring of perceptual resolution by asking participants to identify one blurred face at nine different locations (center, parafoveal, and peripheral) [25]. The results first revealed that the perceptual resolution of participants (measured by threshold levels) decreased with increasing eccentricity and was poorer in the vertical than in the horizontal direction. If participants’ metacognitive monitoring were totally correct, they should guess more at locations with lower perceptual resolution. However, their guess frequency did not show significant difference between vertical and horizontal axes, suggesting an inability to fully distinguish differences in perceptual resolution at the local metacognitive monitoring level. In the global metacognitive monitoring, the results revealed that participants were aware of the overall decrease in resolution as eccentricity increased. Because the perceptual resolution did not reflect an objective task performance (such as accuracy), the difference between the perceptual resolution result and the guess behavior result did not represent the metacognitive monitoring biases. In addition, the participants performance were not identical in the same eccentricity, showing some individuals’ difference. Therefore, it is necessary to adopt a general perceptual paradigm (such as perceptual 2-AFC task) to explore the metacognitive monitoring biases in both local and global level.

Furthermore, the direction of individuals’ monitoring bias regarding their own performance seems to be largely influenced by top-down informational guidance. According to the cue-utilization framework [12, 26], metacognitive judgments draw on both theory-based cues (e.g., self-beliefs, self-concept, self-efficacy) and personal experience, with the former providing top-down guidance [13, 27, 58]. Notably, self-esteem, as an essential component of personality, is closely linked to these theory-based cues [6, 43, 45] and may also influence metacognitive monitoring biases. For example, empirical studies across various domains (e.g., academic performance, interpersonal relationships) indicate that individuals with low self-esteem are more likely to underestiemate their performance globally than their high self-esteem counterparts [2, 6, 34, 40]. A similar phenomenon has been observed at the local level in memory domain [44]. However, research on metacognitive monitoring biases in the perceptual domain based on self-esteem levels is limited. The only relevant study we found divided participants into two groups based on their self-esteem and asked them to complete a perceptual decision-making task, which involved evaluating their overall performance at the end [48]. Their results revealed that, at the global metacognitive monitoring level, individuals with low self-esteem are more likely to underestimate their perceptual performance compared to those with high self-esteem as reflected in their lower subjective ratings. However, their study neither reported information on the local metacognitive monitoring level nor examined the directional biases within the two groups. Therefore, it remains unclear whether self-esteem similarly influences local-level biases, and whether these biases align with those observed at the global level.

In addition, it is crucial to implement strategies to correct metacognitive biases, including reducing the discrepancy between subjective and objective performance and alleviating disparities in monitoring biases across different populations. Feedback appears to be an effective approach in this regard. Previous research has demonstrated that both the provision of accuracy feedback and the valence of feedback can significantly influence metacognitive monitoring. Specifically, providing participants with accuracy feedback has been found to increase their global confidence of performance [47, 48]. In terms of feedback valence, a study on memory found that positive feedback reduces individuals'underestimation bias of their memory abilities, while negative feedback exacerbates it [20]. Similar effects have been observed in perceptual tasks, where global monitoring confidence was higher following positive compared to negative feedback [23]. These findings indicate that both forms of feedback have the potential to affect biases in metacognitive monitoring.

However, when attempting to reduce disparities in metacognitive monitoring biases between individuals with high and low self-esteem, accuracy feedback does not appear to be an effective intervention. Direct evidence comes from a study showing that, even with accuracy feedback, individuals with low self-esteem still underestimated their global performance compared to those with high self-esteem [48]. In contrast, another study examining the effects of feedback valence found that low self-esteem individuals underestimated their value more than high self-esteem individuals in negative feedback conditions, but this disparity disappeared following positive feedback [5]. The findings imply that positive feedback may serve to reduce disparities in monitoring between individuals with high and low self-esteem; however, it did not include a neutral feedback condition to assess the effects of negative feedback. Evidence suggests that individuals with high and low self-esteem differ in the sensitivity to feedback valence [5, 14, 56]. Although there is a general tendency to integrate positive feedback [14, 29, 51], individuals with low self-esteem appear to be more sensitive to negative feedback compared to those with high self-esteem [5, 56]. This difference may reflect the influence of self-congruent feedback [18, 28, 30], whereby individuals tend to prioritize information that aligns with their pre-existing self-beliefs and discount feedback that contradicts these beliefs. Such discrepancies in feedback processing may allow feedback valence to regulate the gap in metacognitive monitoring bias between individuals with high and low self-esteem. Thus, this study mianly focused on how different feedback valences influence the gap of metacognitive monitoring biases across groups.

The present study aimed to address three main questions: (1) the general patterns of local and global metacognitive monitoring biases in the perceptual domain (Experiment 1); (2) whether individuals with low self-esteem exhibit differences in the direction and magnitude of perceptual monitoring biases compared to those with high self-esteem at both local and global levels (Experiment 2); and (3) the effect of feedback valence on metacognitive monitoring biases in individuals with low and high self-esteem.

## Experiment 1

Experiment 1 serves as an initial investigation into the metacognitive monitoring of perceptual ability, primarily exploring whether the monitoring biases could occur, if they exist, the direction of these biases. In addition, we set two levels of difficulty to investigate whether the monitoring bias is a universal phenomenon or only emerge under specific difficulty conditions.

### Method

#### Sample size

In a memory study, Gilbert and colleagues (2020) found that participants consistently underestimated their memory performance at the global level across both Session 1 And Session 2 (Experiment 1). However, Session 2 was administered 14 days after Session 1 to the same group of participants. As the present study does not involve repeated measurements or longitudinal design, the effect size from Session 1 of Experiment 1 (Cohen’s *d* = 0.51), which reflects a single-time-point assessment, was considered more appropriate for our purposes. Therefore, we adopted this effect size to guide our sample size estimation. Based on a power analysis conducted using G*Power 3.1, we determined that a minimum of 33 participants would be required to achieve 80% power to detect a within-group difference with a medium effect size (*d* = 0.51).

#### Participants

A total of 36 undergraduate And graduate participants were recruited online via the WeChat platform And invited to the psychology laboratory to take part in the experiment. After excluding one participant who failed to meet the accuracy threshold of 65%, the final sample included 35 participants (mean age = 22.14, range = 18–26, *SD* = 1.85; 24 females, 11 males).

#### Procedure

**Fig. 1:**
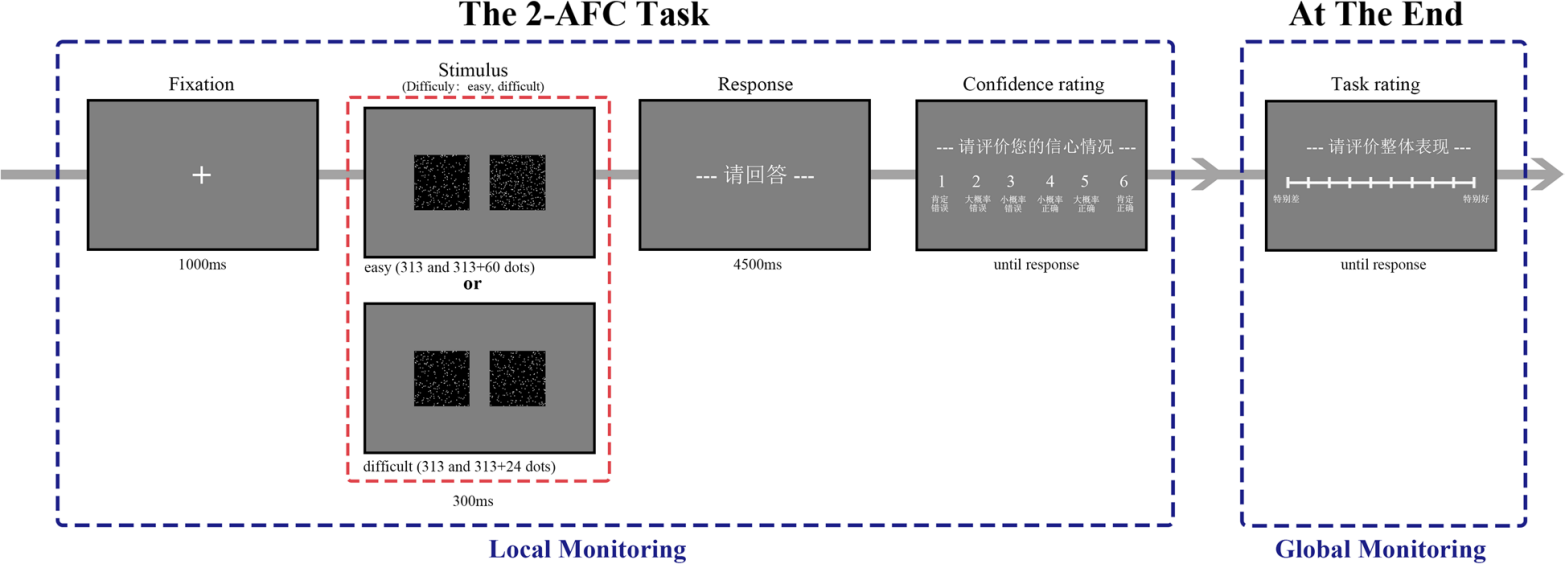
Trial procedure of Experiment 1. The practice and main phases of the experiment followed the same procedure, with the only difference being the number of trials

This study employed a perceptual 2-AFC task with confidence ratings, a well-established paradigm for assessing metacognitive processes in visual perception [16, 47], with the procedure detailed in Fig. 1. In the task, participants were required to determine which of two boxes contained more dots and respond using a button press. Each trial began with the presentation of a white fixation point (viewing distance: approximately 57 cm, visual Angle: 1° × 1°) for 1000 ms. This was followed by the display of two black squares, one on each side of the screen (visual Angle: 18.9° × 7.4°), for 300 ms. Each square contained varying numbers of white dots: one box consistently held 313 dots (half-filled with 625 possible positions), while the other contained either 313 + 24 dots (difficult condition) or 313 + 60 dots (easy condition) [48].

Participants were prompted to respond when the text"Please answer"appeared on the screen. They were instructed to press the"1"key if the left square contained more dots and the"6"key if the right square contained more. The correct location of the greater number of dots was evenly distributed between the left And right sides. Responses were required within 4500 ms. After each response, participants rated their confidence in their answer's accuracy on a scale from 1 to 6, where 1 represented"definitely wrong (肯定错误),"2"probably wrong (大概率错误),"3"possibly wrong (小概率错误),"4"possibly right (小概率正确),"5"probably right (大概率正确),"and 6"definitely right (肯定正确)."

At the end of the experiment, participants provided An overall task performance rating on a scale from 1 to 10, with 1 indicating"terrible"performance And 10 indicating"excellent,"while intermediate points represented varying degrees of evaluation.

The current experiment consisted of a practice phase And a main phase. During the practice phase, each difficulty level was presented randomly 3 times, resulting in a total of 6 trials. In the main phase, each difficulty level was presented randomly 80 times, for a total of 160 trials.

#### Measures

##### Local monitoring bias

The bias was defined as the absolute discrepancy between performance on the perceptual task (correct or incorrect judgment of which box contained more dots) and the corresponding confidence judgment [11]. For correct judgments, the confidence rating of"definitely right"represented the most appropriate assessment, resulting in a discrepancy of zero. Ratings of"probably right","possibly right","possibly wrong","probably wrong", and"definitely wrong"corresponded to discrepancies of −1, −2, −3, −4, and −5, respectively, reflecting increasing underconfidence in performance. In contrast, for incorrect judgments, the confidence rating of"definitely wrong"was considered the most accurate, resulting in a discrepancy of zero. Ratings of"probably wrong","possibly wrong","possibly right","probably right", and"definitely right"corresponded to discrepancies of + 1, + 2, + 3, + 4, and + 5, respectively, reflecting increasing overconfidence. The mean discrepancy across all trials was calculated for each participant to serve as an index of monitoring bias. This index ranged from −5, indicating maximum performance underestimation, to + 5, indicating maximum performance overestimation, with scores near zero reflecting accurate local metacognitive monitoring.

##### Global monitoring bias

The bias was measured by the difference between the overall task accuracy And the transformed post-task ratings. The original post-task ratings ranged continuously from 0 to 10, while the transformed score was adjusted to fall within the 0 to 1 range. The global monitoring bias was calculated by subtracting the overall task accuracy from the transformed post-task ratings. This value ranged from −1, indicating maximal performance underestimation, to 1, indicating maximal performance overestimation, with values near zero reflecting accurate global metacognitive monitoring without bias.

#### Data analysis

In this experiment, the independent variable was task difficulty (easy vs. difficult), while the dependent variables included task accuracy, local And global metacognitive monitoring bias. All Analyses were conducted using IBM SPSS Statistics 27.

### Results

**Fig. 2:**
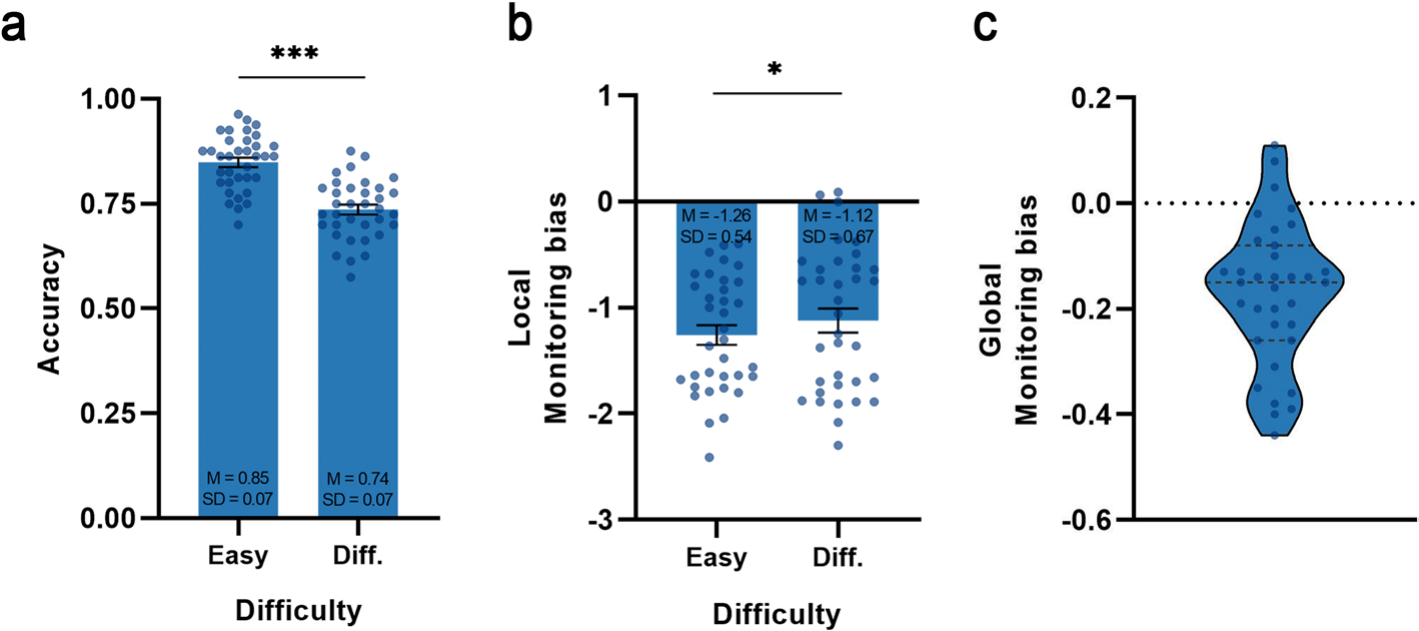
Behavioral results from Experiment 1. **a** The task accuracy across varying levels of difficulty. **b** The local monitoring bias value between easy and difficulty task. **c** The global monitoring bias value, with the three horizontal lines from top to bottom representing the third quartile (Q3), the mean, and the first quartile (Q1), respectively. In all figures, the error bars represent SEMs across participants, the dots in figure **a** and **c** indicate individual data points. *p <.05, **p <.01, ***p <.001

We first analyzed the overall task performance (Fig. 2a), paired-samples t-tests revealed that accuracy was significantly higher in the easy condition compared to the difficult condition, *t*(34) = 9.918, *p* < 0.001, Cohen’s *d* = 1.676, 95% CI = [0.09, 0.14].

Next, we investigated whether there was a significant metacognitive monitoring bias in perceptual ability. As for local monitoring bias (Fig. 2b), one-sample t-tests revealed that the bias values were significantly less than 0 under the easy task condition, *t*(34) = −13.658, *p* < 0.001, Cohen’s *d* = −2.309, 95% CI = [−1.44, −1.07], the difficulty conditions,*t*(34) = −9.899, *p* < 0.001, Cohen’s *d* = −1.673, 95% CI = [−1.35, −0.89], and the overall condition, *t*(34) = −12.072, *p* < 0.001, Cohen’s *d* = −2.041, 95% CI = [−1.39, −0.99]. And the negative local monitoring bias value was significantly lower in the easy condition than in the difficult condition, *t*(34) = −2.239, *p* = 0.032, Cohen’s *d* = −0.379, 95% CI = [−0.26, −0.01]. As for global monitoring bias (Fig. 2c), one-sample t-tests also revealed that the bias values were significantly less than 0, *t*(34) = −7.401, *p* < 0.001, Cohen’s *d* = −1.251, 95% CI = [−0.22, −0.12].

#### Discussion

The current findings demonstrate that both local and global metacognitive monitoring biases were consistently and significantly below zero. Furthermore, local monitoring bias remained significantly negative across perceptual tasks of varying difficulty. These results suggest a consistent tendency among participants to underestimate their perceptual performance, irrespective of task difficulty.

Of course, it should be noted that the current local metacognitive monitoring bias is actually calculated by the difference between the true accuracy and participants’ confidence about their performance. As such, the proportion of correct and incorrect trials may influence the extent of the bias (see Supplementary Materials). The detail discussion can been seen in the general discussion section.

## Experiment 2

The results of Experiment 1 revealed a consistent tendency for individuals to underestimate their perceptual perfomance during metacognitive monitoring. In Experiment 2, we aimed to investigate the differences in the direction and magnitude of global and local monitoring biases between individuals with high and low self-esteem. It was hypothesized that individuals with low self-esteem would show a more pronounced degree of underestimation in both local and global monitoring.

In Experiment 2, we still retained two levels of difficulty. Although Experiment 1 demonstrated that local monitoring bias is more pronounced under easier conditions, it remains possible that such conditions could lead to similarly large local monitoring biases for both high and low self-esteem groups, thereby masking the differential effect (reaching to a ceiling effect).

### Method

#### Recruitment criteria

The Rosenberg Self-Esteem Scale (RSES; [46]) was employed to assess self-esteem levels within the population. The questionnaires were distributed via An online survey tool, resulting in the collection of 248 valid responses. Based on the distribution of scores, Q1 (27) and Q3 (32) were defined as key thresholds for categorizing participants. Specifically, individuals scoring below Q1 were categorized as low self-esteem participants, while those scoring above Q3 were categorized as high self-esteem participants. Individuals meeting these criteria were subsequently invited to participate in the follow-up experiment.

#### Sample size

A previous study examining self-esteem And metacognitive monitoring in the context of perceptual performance recommended recruiting more than 30 participants per group to ensure adequate statistical power [48]. This guideline was adopted in the current study.

#### Participants

A total of 64 undergraduate And graduate participants were recruited online via the WeChat platform And invited to the psychology laboratory to take part in the experiment. After excluding 2 participants who did not meet the required accuracy threshold of 65%, the final sample consisted of 62 valid participants (mean age = 21.21, range = 18–26, *SD* = 2.06; 47 females, 15 males), with 31 participants in both the low and high self-esteem groups.

#### Procedure

Based on the online RESE questionnaire, participants who met the criteria were invited to the laboratory to complete the experimental task within two days of completing the questionnaire. The task was identical to that used in Experiment 1.

#### Data analysis

The behavioral experimental task was conducted in accordance with the procedure employed in Experiment 1. This experiment utilized a 2 × 2 mixed design, with self-esteem (high vs. low) as the between-subjects variable and task difficulty (easy vs. difficult) as the within-subjects variable. The dependent variables included task accuracy, local monitoring bias, global monitoring bias And metacognitive efficiency. All Analyses were conducted using IBM SPSS Statistics 27.

### Results

We first examined whether there were any differences in the direction of local and global monitoring bias between participants with high and low self-esteem. For high self-esteem group, one-sample t-tests demonstrated that, across a range of perceptual tasks of varying difficulty, the local monitoring bias values were significantly less than zero (easy: *t*(30) = −9.379, *p* < 0.001, Conhen’s *d* = −1.685, 95% CI = [− 1.17, − 0.75]; difficult: *t*(30) = −5.631, *p* < 0.001, Conhen’s *d* = −1.011, 95% CI = [− 1.01, − 0.47]; overall: *t*(30) = −7.598, *p* < 0.001, Conhen’s *d* = −1.365, 95% CI = [− 1.08, − 0.62]). In addition, the value of global monitoring bias also remained significantly negative, *t*(30) = −4.502, *p* < 0.001, Conhen’s *d* = −0.809, 95% CI = [− 0.16, − 0.06]. For the low self-esteem group, one-sample t-tests revealed that the local monitoring bias for the low self-esteem group was significantly lower than zero (easy: *t*(30) = −12.779, *p* < 0.001, Conhen’s *d* = −2.295, 95% CI = [− 1.61, − 1.17]; difficult: *t*(30) = −7.626, *p* < 0.001, Conhen’s *d* = −1.370, 95% CI = [− 1.33, − 0.77]; overall: *t*(30) = −10.397, *p* < 0.001, Conhen’s *d* = −1.867, 95% CI = [− 1.46, − 0.98]). Additionally, the value of global monitoring bias was also significantly negative, *t*(30) = −8.035, *p* < 0.001, Conhen’s *d* = −1.443, 95% CI = [− 0.25, − 0.15]. This finding aligns with the results of Experiment 1, indicating consistency in the direction of monitoring bias for both high and low self-esteem participants when evaluating their perceptual performance.

**Fig. 3:**
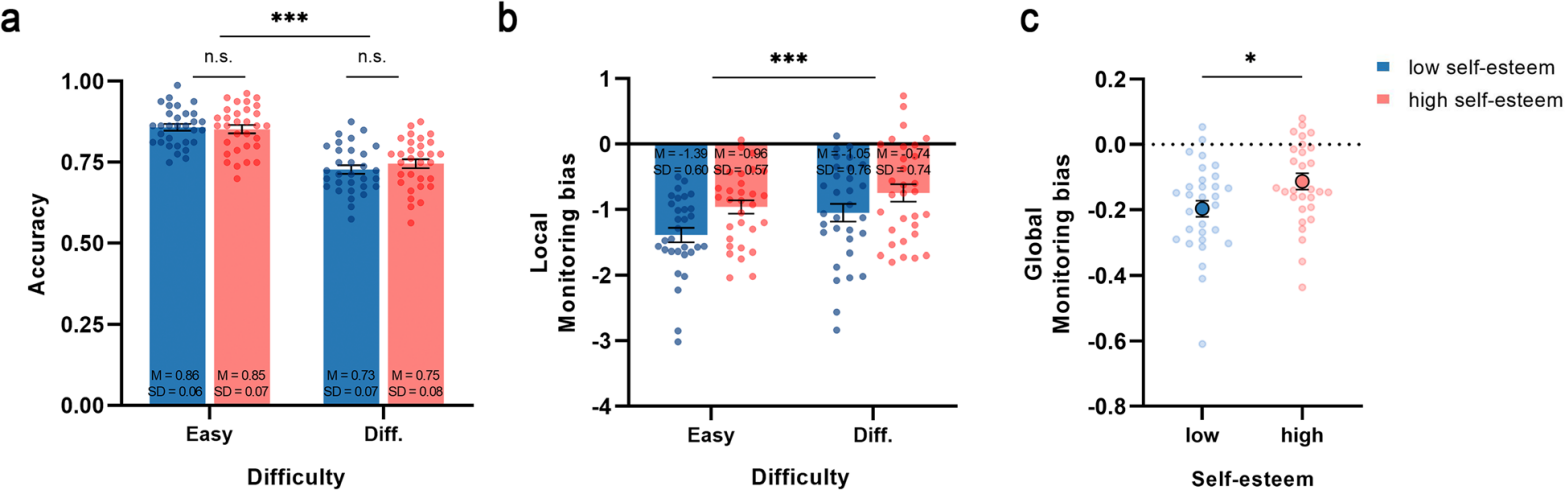
Behavioral results from Experiment 2. **a** The task accuracy of individuals with high and low self-esteem across varying levels of difficulty. **b** The local monitoring bias value between individuals with high and low self-esteem in easy and difficulty task. **c** The global monitoring bias value between individuals with high and low self-esteem. In all figures, the error bars represent SEMs across participants, the dots in figure indicate individual data points. *p <.05, **p <.01, ***p <.001

Next, a 2 × 2 mixed-design ANOVA on objective task accuracy revealed a significant main effect of task difficulty (Fig. 3a), *F*(1,60) = 167.962, *p* < 0.001, *η*_*p*_^2^ = 0.737. However, there was no significant main effect of self-esteem, *F*(1,60) = 0.164, *p* = 0.687, *η*_*p*_^2^ = 0.003, nor a significant interaction between task difficulty and self-esteem, *F*(1,60) = 1.697, *p* = 0.198, *η*_*p*_^2^ = 0.028. This indicates that there are no statistically significant differences in objective task performance between participants with low and high self-esteem.

Finally, to investigate differences in the extent of global and local metacognitive monitoring bias between high And low self-esteem participants, a series of Analyses were conducted on local monitoring bias and global monitoring bias. As for local monitoring bias, a 2× 2 mixed-design ANOVA revealed a significant main effect of self-esteem (Fig. 3b), *F*(1,60) = 5.111, *p* = 0.027, *η*_*p*_^2^ = 0.078, with the low self-esteem group (*M* = −1.22, *SD* = 0.65) consistently demonstrating a greater negative monitoring bias compared to the high self-esteem group (*M* = −0.85, *SD* = 0.62). Additionally, the main effect of difficulty was significant, *F*(1,60) = 25.488, *p* < 0.001, *η*_*p*_^2^ = 0.298. Specifically, local monitoring bias was more negative in the easy condition than in the difficult condition, consistent with the findings from Experiment 1. No significant interaction effect was observed, *F*(1,60) = 1.382, *p* = 0.244, *η*_*p*_^2^ = 0.023.

As for global monitoring bias (Fig. 3c), the results of the independent samples t-tests indicated the bias value were significantly lower in the group with low self-esteem compared to the group with high self-esteem, *t*(60) = −2.390, *p* = 0.020, Cohen’s *d* = −0.607, 95% CI = [− 0.15, − 0.01]. This finding indicates that individuals with low self-esteem demonstrate a greater underestimation tendency in global metacognitive monitoring.

#### Discussion

The results of Experiment 2 revealed that both high And low self-esteem participants exhibited local And global monitoring biases in perceptual performance that were significantly below zero, indicating consistency across the two groups. This finding aligns with those of Experiment 1, suggesting that participants, irrespective of their self-esteem levels, tend to underestimate their perceptual performance.

Further analysis revealed no significant difference in perceptual task accuracy between the high and low self-esteem groups, suggesting comparable perceptual abilities at an objective level. However, at the metacognitive level, low self-esteem individuals exhibited a more pronounced underestimation bias than their high counterparts, evident not only in global monitoring but also in local monitoring across both easy and difficult tasks.

## Experiment 3

The results in Experiment 2 have revealed individuals with low self-esteem exhibited more pronounced underestimation monitoring bias than those with high self-esteem, both on local And global monitoring process. In Experiment 3, we aimed to investigate the effects of feedback valence on local and global monitoring biases among individuals with high versus low self-esteem. To examine the independent effects of positive and negative feedback, a neutral feedback condition was introduced as a baseline.

Prior research suggests that feedback valence may influence metacognitive monitoring by altering individuals’ self-beliefs [20, 23], and individuals tend to intergrate positive feedback [28, 30, 51]. Thus, we hypothesized that positive feedback may reinforce indiviruals’ self-beliefs, then the underestimation monitoring bias should be diminished. On the other hand, the regulatory intensity may be also modulated by the self-congruent feedback. Specifically, the negative feedback is more consistent with the beliefs of low self-esteem individuals [15, 45] and may thus be more readily internalized, further weakening their beliefs. But the high-esteem usually has a positive self-belief [3, 6], which conflicts the negative feedback. Consequently, we hypothesized that negative feedback would exert a greater impact on low self-esteem individuals than on high self-esteem individuals.

### Method

#### Recruitment criteria

The recruitment criteria were identical to those used in Experiment 2.

#### Sample size

A power analysis conducted using G*Power 3.1 indicated that a minimum of 24 participants per group would be required to achieve 80% power to detect a 2 × 3 within-between interaction effect with a medium effect size (*f* = 0.25). This effect size aligns with findings from a related study on the impact of feedback valence on self-esteem (*η*_*p*_^2^ = 0.06; [5]). To ensure the reliability of the results, the sample size for each group was increased to at least 30 participants.

#### Participants

A total of 74 undergraduate And graduate participants were recruited online via the WeChat platform And invited to the psychology laboratory to take part in the experiment. After excluding 2 participants who did not meet the required accuracy threshold of 65%, the final sample consisted of 72 valid participants (mean age = 21.81, range = 18–29, *SD* = 2.48; 59 females, 13 males), with 36 participants in both the low and high self-esteem groups.

#### Procedure

The participants in both the high self-esteem and the low self-esteem groups were required to complete four sessions, with each session consisting of a practice phase and a experiment phase. In the experiment phase of all the sessions, a task of medium difficulty was employed (one box was consistently half-filled, while the other box contained 313 + 42 dots). However, the difficulty of the tasks in the practice phase and the feedback provided during the experiment phase differed.

Participants were assigned randomly to two groups, each with 18 high self-esteem And 18 low self-esteem individuals. The experiment comprised four sessions: in Sessions 2 And 4, both groups completed a medium-difficulty task during the practice phase And received objective feedback in the experiment phase. In Session 1, Group 1 performed An easy task And received positive feedback, while Group 2 performed a difficult task And received negative feedback; Session 3 reversed these conditions (for a detailed description of the feedback, see Table 1; for the session sequence, see Table 2).

**Table 1:** Feedback provided in the objective, positive and negative conditions

Accuracy	Feedback (objective condition)	Feedback (positive condition)	Feedback (negative condition)
0%	Your accuracy is 0 (您的正确率为0)	No correct answers yet—keep going! (暂无正确, 继续加油!)	All incorrect—room for improvement! (全部错误, 需要努力!)
0–50%	Your accuracy is between 0 And 50% (您的正确率在0和50%之间)	Basically correct! Your performance has met our expectations! (基本正确!您的表现达到了我们的预期!)	Most answers are incorrect! Your performance did not meet our expectations! (多数错误!您的表现未满足我们的预期!)
50–100%	Your accuracy is between 50 And 100% (您的正确率在50%和100%之间)	Mostly correct! Your performance has exceeded our expectations! (多数正确!您的表现超过了我们的预期!)	There are still errors! Your performance did not meet our expectations! (仍有错误!您的表现未达到我们的预期!)
100%	Your accuracy is 100% (您的正确率为100%)	All correct! Your performance has exceeded our expectations! (全部正确!您的表现超过了我们的预期!)	No errors—maintain this level of performance! (没有错误, 需要继续保持!)

**Table 2:** Session sequence for Group 1 And Group 2

Session	Group 1		Group 2	
	Practice phase	Experiment phase	Practice phase	Experiment phase
1	easy	positive	difficult	negative
2	medium	objective	medium	objective
3	difficult	negative	easy	positive
4	medium	objective	medium	objective

The "Practice phase" column indicates the task difficulty level during the practice phase, while the "Experiment phase" column specifies the type of feedback provided in the experiment phase

Thus, each participant was exposed to three distinct conditions across the four sessions: a difficult practice followed by negative feedback, a simple practice followed by positive feedback, and a moderate difficulty practice followed by objective feedback. It is reasonable to assume that participants would experience a negative priming effect when confronted with a difficult task during the practice phase. If they subsequently received negative feedback during the experiment phase, this may further amplify the impact of negative valence. Conversely, encountering an easy task during the practice phase may induce a positive priming effect, such that subsequent positive feedback in the experiment phase could enhance the influence of positive valence. When participants faced tasks of moderate difficulty in the practice phase and received objective feedback in the experiment phase, this can be regarded as a baseline condition.

**Fig. 4:**
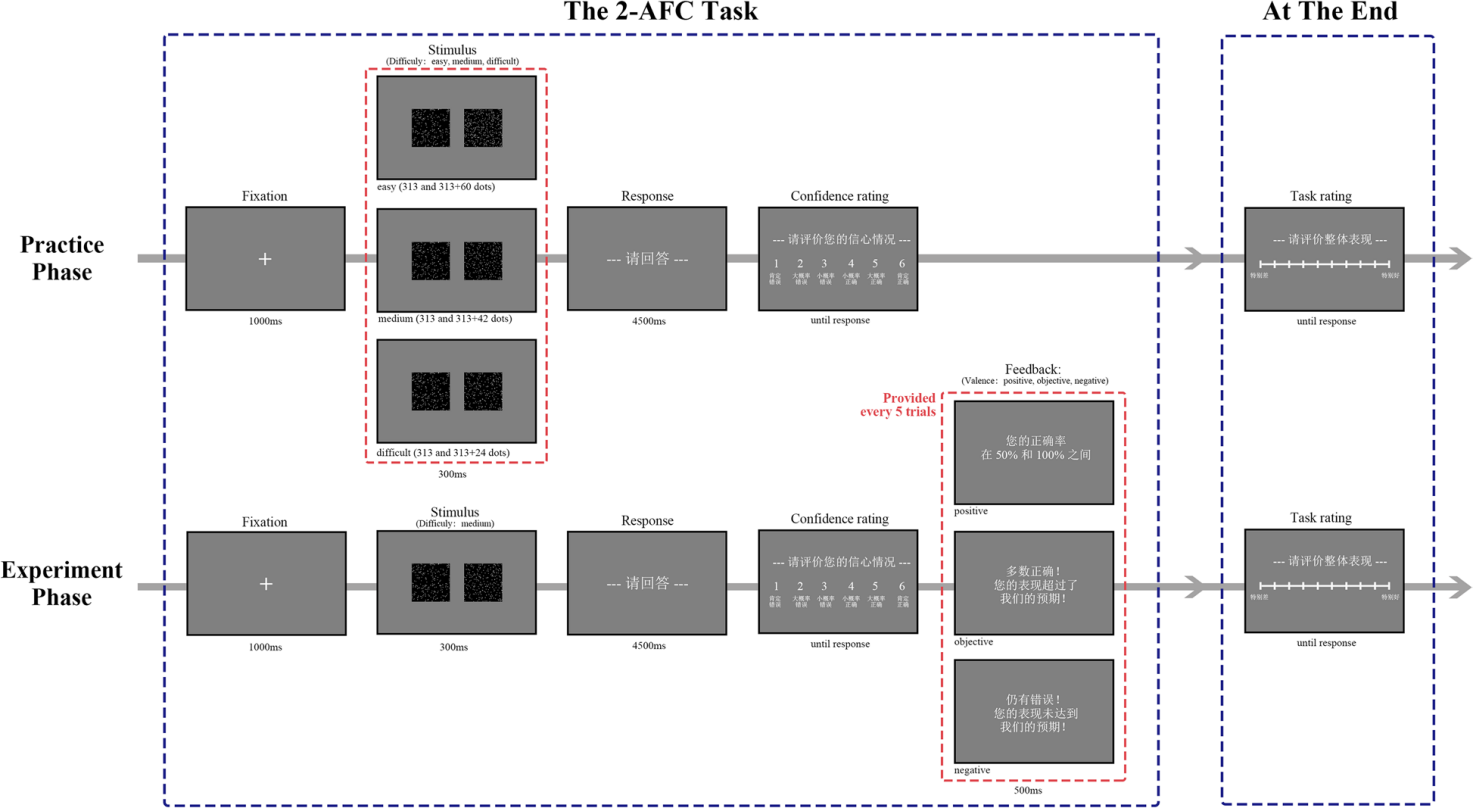
Trial procedure of Experiment 3

Each session’s practice phase consisted of 32 trials. The experiment phase included 96 trials for both the positive And negative feedback sessions, And 64 trials for the objective feedback session. Since the objective feedback condition was repeated twice (i.e., two sessions), its number of experiment-phase trials was reduced by one-third to keep the overall experiment duration manageable. Feedback was provided every five trials in experiment phase (for a detailed experimental procedure, see Fig. 4).

#### Data analysis

This experiment utilized a 2 × 3 mixed design, with self-esteem (high vs. low) as the between-subjects variable and difficulty-feedback (positive vs. negative vs. objective) as the within-subjects variable. The dependent variables included task accuracy, local And global metacognitive monitoring bias. All Analyses were conducted using IBM SPSS Statistics 27.

### Results

Firstly, we investigated whether feedback valence affected the direction of monitoring bias. For the low self-esteem group, one-sample t-tests revealed that, across different conditions, the local monitoring bias values for both groups was significantly less than 0 (positive: *t*(35) = −6.069, *p* < 0.001, Conhen’s *d* = −1.012, 95% CI = [− 0.97, − 0.49]; objective: *t*(35) = −6.515, *p* < 0.001, Conhen’s *d* = −1.086, 95% CI = [− 1.16, − 0.61]; negative: *t*(35) = −7.450, *p* < 0.001, Conhen’s *d* = −1.242, 95% CI = [− 1.37, − 0.78]). In addition, the global monitoring bias values remained significantly negative as well (positive: *t*(35) = −2.376, *p* = 0.023, Conhen’s *d* = −0.396, 95% CI = [− 0.11, − 0.01]; objective: *t*(35) = −5.807, *p* < 0.001, Conhen’s *d* = −0.968, 95% CI = [− 0.19, − 0.09]; negative: *t*(35) = −11.530, *p* < 0.001, Conhen’s *d* = −1.922, 95% CI = [− 0.31, − 0.21]). For the high self-esteem group, one-sample t-tests revealed that the local monitoring bias values for the the high self-esteem group was significantly lower than zero (postive: *t*(35) = −4.285, *p* < 0.001, Conhen’s *d* = −0.714, 95% CI = [− 0.72, − 0.26]; objective: *t*(35) = −4.561, *p* < 0.001, Conhen’s *d* = −0.760, 95% CI = [− 0.70, − 0.27]; negative: *t*(35) = −4.826, *p* < 0.001, Conhen’s *d* = −0.804, 95% CI = [− 0.73, − 0.30]). In contrast, the global monitoring bias values showed no significant difference from zero in the positive condition, but remained significantly lower than zero in both the negative and objective conditions (postive: *t*(35) = −1.002, *p* = 0.323, Conhen’s *d* = −0.167, 95% CI = [− 0.08, 0.03]; objective: *t*(35) = −5.636, *p* < 0.001, Conhen’s *d* = −1.728, 95% CI = [− 0.16, − 0.07]; negative: *t*(35) = −10.370, *p* < 0.001, Conhen’s *d* = −1.728, 95% CI = [− 0.29, − 0.19]).

**Fig. 5:**
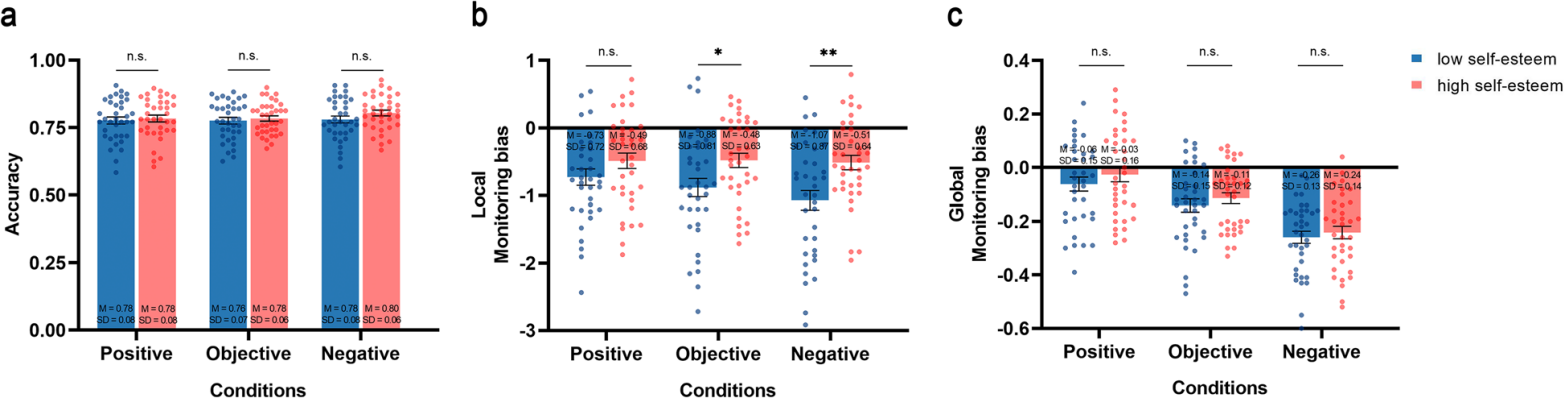
Behavioral results from Experiment 3. **a** The task accuracy of individuals with high and low self-esteem across varying feedback conditions. **b** The local monitoring bias value between individuals with high and low self-esteem across varying feedback conditions. **c** The global monitoring bias value between individuals with high and low self-esteem across varying feedback conditions. In all figures, the error bars represent SEMs across participants, the dots in figure a indicate individual data points. *p <.05, **p <.01, ***p <.001

Next, a 2 × 3 mixed-design ANOVA was performed to assess the accuracy of the task completed by participants (Fig. 5a). The analysis revealed no significant main effect of feedback, *F*(2,140) = 1.863, *p* = 0.159, *η*_*p*_^2^ = 0.026, nor of self-esteem, *F*(1, 70) = 0.804, *p* = 0.373, *η*_*p*_^2^ = 0.011. The interaction between feedback and self-esteem was also non-significant, *F*(2,140) = 0.781, *p* = 0.460, *η*_*p*_^2^ = 0.011. These results indicate that there were no differences in objective accuracy between the two groups across the different conditions.

Finally, we investigated the impact of feedback conditions on the gap in local and global metacognitive monitoring bias between individuals with high and low self-esteem (Fig. 5b). As for local monitoring bias, a 2 × 3 mixed-design ANOVA conducted on local monitoring bias revealed a significant main effect of feedback valence, *F*(2,140) = 5.023, *p* = 0.008, *η*_*p*_^2^ = 0.067. The effect of self-esteem reached significance as well, *F*(1,70) = 6.410, *p* = 0.014, *η*_*p*_^2^ = 0.084, with the low self-esteem group (*M* = −0.90, *SD* = 0.75) consistently demonstrating a lower monitoring bias value compared to the high self-esteem group (*M* = −0.49, *SD* = 0.58). The interaction effect also reached significance, *F*(2,140) = 3.690, *p* = 0.027, *η*_*p*_^2^ = 0.050. A simple effects analysis (Bonferroni) revealed that different feedback conditions had a significant impact on the value of local metacognitive monitoring bias in the low self-esteem group, *F*(2,69) = 7.235, *p* = 0.001, *η*_*p*_^2^ = 0.173, whereas no significant differences in local monitoring bias were found across the three conditions for the high self-esteem group (*F*(2,69) = 0.116, *p* = 0.890, *η*_*p*_^2^ = 0.003). Notably, in both the negative and objective conditions, the low self-esteem group demonstrated a lower bias value compared to the high self-esteem group (negative: *F*(1,70) = 9.760, *p* = 0.003, *η*_*p*_^2^ = 0.122; objective: *F*(1,70) = 5.487, *p* = 0.022, *η*_*p*_^2^ = 0.073). In contrast, no significant differences in local monitoring bias were observed between the two groups in the positive condition (*F*(1,70) = 2.107, *p* = 0.151, *η*_*p*_^2^ = 0.029).

As for global monitoring bias, a 2 × 3 mixed-design ANOVA conducted on global monitoring bias revealed a significant main effect of feedback conditions, *F*(2,140) = 68.699, *p* < 0.001, *η*_*p*_^2^ = 0.495. Specifically, the values of global monitoring bias were lowest in the negative condition (*M* = −0.25, *SD* = 0.14), highest in the positive condition (*M* = −0.04, *SD* = 0.16), with the objective condition (*M* = −0.13, *SD* = 0.13) yielding ratings in between. No significant main effect of self-esteem was observed, *F*(1,70) = 0.961, *p* = 0.330, *η*_*p*_^2^ = 0.14. The interaction effect did not reach significance as well, *F*(2,140) = 0.118, *p* = 0.889, *η*_*p*_^2^ = 0.002.

#### Discussion

The results of Experiment 3 indicate that the local monitoring bias values were consistently below zero for both self-esteem groups, suggesting a stable tendency to underestimate perceptual performance, regardless of the feedback valence, consistent with Experiments 1 And 2. In contrast, for the high self-esteem group, global monitoring bias was significantly negative only in the objective and negative conditions but approached zero in the positive condition, suggesting that the global monitoring process in individuals with high self-esteem may be more accurate when positive feedback is provided.

Furthermore, for local metacognitive monitoring, the main effect of feedback valence was significant, indicating that positive feedback effectively reduced the monitoring bias gap between individuals with high and low self-esteem, whereas negative feedback amplified this gap. Notably, feedback significantly influenced local monitoring bias only in the low self-esteem group, with no significant changes observed across feedback conditions in the high self-esteem group. This indicates that individuals with low self-esteem may be more sensitive to the effects of feedback valence on local monitoring bias than their high self-esteem counterparts, which will be further discussed in the General Discussion section. For global metacognitive monitoring, no significant differences in monitoring bias were found between high and low self-esteem groups across feedback conditions, suggesting that feedback—regardless of valence—may help reduce differences based on self-esteem in global monitoring. Moreover, unlike its differential effects on local monitoring, feedback valence had a similar impact on global monitoring bias across both groups. Specifically, the strongest underestimation bias occurred under negative feedback, and the weakest under positive feedback, indicating that global monitoring may be more influenced by feedback valence than by self-esteem.

## General discussion

This study, which integrated a perceptual 2-AFC task with confidence ratings, revealed that individuals tended to underestimate their perceptual performance in both local and global metacognitive monitoring, reflecting a general uncertainty about their performance (Experiment 1–3). These findings are consistent with studies in the memory domain, which report a similar underconfidence of memory abilities [20, 49]. Given the close relationship between metacognitive processes in perception and memory—such as near-transfer effects [7] and shared neural networks (Vaccaro & Fleming, 2018)—it is possible that the underestimation bias is more likely observed in the fundermental cognitive processes. Additionally, we observed that local monitoring bias was significantly greater in the easy condition than in the difficult condition. This effect may be driven by two factors. First, lower task demands may lead to reduced cognitive engagement, which has been shown to impair performance [17, 59]. Such reductions in effort may similarly impair metacognitive monitoring. Second, the magnitude of local monitoring bias may be influenced by the relative proportion of correct and incorrect trials (see Supplementary Materials). Since the easy condition generally produces more correct responses and fewer errors, this imbalance may lead to a more negative local monitoring bias. Therefore, it may be valuable for future studies to consider the potential influence of trial-type distribution when interpreting monitoring bias.

A previous study has shown that individuals with low self-esteem tend to underestimate their perceptual performance at the global level compared to those with high self-esteem [48]. The current study further examined both global and local monitoring, finding that low self-esteem individuals showed pronounced underestimation bias at both levels (Experiment 2). This finding aligns with research across various domains showing that individuals with low self-esteem tend to hold more negative self-evaluations [3, 44, 58], a pattern that is thought to stem from their internalized negative self-beliefs [41, 45]. According to Fennell’s model of low self-esteem behavior, individuals with low self-esteem often develop pervasive negative core beliefs [15, 45], which was suggested to influence metacognitive judgement [13, 58]. Specifically, the negative self-beliefs held by individuals with low self-esteem may lead them to adopt a more pessimistic stance when making metacognitive judgments. At the global level, individuals with low self-esteem may perceive their overall task performance as poor due to their negative self-beliefs, even when their actual performance is adequate. At the local level, these beliefs may lead to reduced confidence in the correctness of their individual responses, regardless of objective accuracy. Therefore, the greater underestimation bias observed in individuals with low self-esteem may also be driven by the influence of their negative self-beliefs on metacognitive judgment.

Additionally, it might argue that the low self-esteem has the difficulties in distinguishing correct from incorrect perceptual judgments compared to the high self-esteem, which might contribute to the greater underestimation bias in the low self-esteem. As a measure of the ability to differentiate between correct and incorrect judgments (based on signal detection theory), the metacognitive efficiency provides an effective indicator for testing this hypothesis [36]. Previous research suggests that low self-esteem individuals have lower confidence [3, 10], and lower confidence is correlated with reduced metacognitive efficiency [52, 57]. Thus, low self-esteem individuals may exhibit lower metacognitive efficiency. However, we calculated the metacognitive efficiency for both high and low self-esteem groups (see supplementary materials) and found no significant differences, indicating comparable abilities to distinguish between correct and incorrect responses. Therefore, by demonstrating that the two groups exhibited similar levels of metacognitive efficiency, we were able to reasonably exclude differences in metacognitive ability as the explanation for the observed bias.

For local monitoring, negative feedback increased underestimation in the low self-esteem group but had no significant effect on the high self-esteem group. These findings are consistent with previous research showing that low self-esteem individuals are more sensitive to negative feedback [56], whereas high self-esteem may buffer against its impact [5]. This might highlight the role of feedback congruence, with individuals tending to accept feedback that aligns with their pre-existing self-beliefs and reject feedback that contradicts it [14],García‐Arch et al., 2024; [39]. Low self-esteem individuals may be more likely to integrate negative feedback that aligns with their typical negative self-beliefs [15, 45], which might reinforce their negative self-beliefs and exacerbate their metacognitive underestimation bias. In contrast, high self-esteem individuals may dismiss negative feedback that conflicts with their positive self-beliefs [3, 6], thereby limiting its effect on metacognitive monitoring. However, although positive feedback contradicts the negative self-beliefs of low self-esteem individuals, it still alleviated their underestimation bias, which may highligt the role of positivity bias. Namely, individuals tend to integrate positive feedback to maintain a favorable view of the self [29, 51]. Thus, low self-esteem individuals may accept the positive feedback, which in turn helped mitigate their negative self-beliefs and reduced the extent of their underestimation bias. These findings suggest that, at the local level, positivity bias and feedback congruency may jointly influence how feedback valence affects monitoring bias in low self-esteem individuals.

However, the effects of positivity bias And self-congruency on the local monitoring biases of individuals with high self-esteem require further investigation. Results from Experiment 3 showed that positive feedback did not significantly influence the local monitoring bias in high self-esteem individuals. Several potential explanations may account for this outcome. First, individuals with high self-esteem may already hold strong positive self-beliefs, leaving limited room for positive feedback to further enhance it, thereby minimizing its impact on their metacognitive monitoring bias. Secondly, the positive feedback may have been less impactful than intended, as written text alone might lack sufficient emotional salience. Future studies could employ more emotionally evocative feedback, such as images, facial expressions, or emojis, to better evaluate their potential effects. Therefore, future research could directly assess self-beliefs and optimize feedback procedures to better understand their role in metacognitive monitoring biases.

For the global monitoring process, we observed an inconsistency with the trend of local monitoring bias changes, as no significant differences in monitoring bias were found between low and high self-esteem individuals across all three conditions. A possibility might be that differences in information sources between global and local monitoring lead to their varying sensitivity to feedback valence [19, 21]. Local metacognitive monitoring, which focus on confidence judgments made in real time, relies more heavily on ‘bottom-up’ cues derived from trial-by-trial performance. In contrast, global monitoring occurs retrospectively following task completion and is thought to incorporate both prior experiences and self-perceptions of ability [21]. Within this context, feedback serves as a salient and explicit cue, which the global monitoring process may weight more heavily. These differences in underlying mechanisms may account for the stronger effects of feedback observed in global monitoring than self-esteem levels.

In addition, it should be noted that the sample used in this study was drawn from China; as such, the findings primarily reflect the characteristics embedded within the Chinese cultural context. Previous research has demonstrated that cultural differences significantly influence feedback processing [28, 30, 32]. For instance, Korn et al. [28, 30] found that Chinese participants were more susceptible to personality feedback than German participants, with distinct medial prefrontal cortex activity during feedback processing. These results suggest that culture may modulate the psychological and neural mechanisms underlying feedback. Therefore, future research should examine whether the present findings hold across diverse cultural contexts to assess their generalizability.

These findings highlight the importance of attending to students’ self-esteem in educational contexts. For learners with low self-esteem, the use of supportive strategies and positive feedback may help reduce their tendency to underestimate their performance. In contrast, negative feedback should be delivered with caution, as it may exacerbate metacognitive underestimation in individuals with low self-esteem. While students with high self-esteem are generally more resilient to negative input, a balanced feedback approach remains essential, as excessive negative feedback may still undermine their global metacognitive monitoring.

## Conclusion

Across three experiments, the present study revealed that individuals tend to underestimate their perceptual performance at both the local and global levels, with this underestimation bias being more pronounced in individuals with low self-esteem compared to those with high self-esteem. Importantly, positive feedback alleviated monitoring biases in both local and global metacognitive monitoring for low self-esteem individuals, while negative feedback exacerbated these biases. In contrast, for high self-esteem individuals, this effect was observed only at the global level. Additionally, feedback valence was found to reduce the disparity in global monitoring biases between high and low self-esteem individuals, whereas only positive feedback alleviated the difference in local monitoring. These findings suggest that the influence of feedback valence on metacognitive monitoring biases varies depending on both self-esteem level and the level of monitoring.

## Supplementary Information

Supplementary Material 1.

Supplementary Material 2.

## Data Availability

Data is provided within the manuscript or supplementary information files.
