# Supplementary material for: Perceptual metacognition and self-esteem: the role of feedback valence in local and global monitoring bias
Source: BMC Psychol. 2025 Sep 22;13:1114. doi: 10.1186/s40359-025-03089-x (PMC12506185; doi:10.1186/s40359-025-03089-x)
Supplement: Supplementary file 2 — Supplementary Material 2. [file 40359_2025_3089_MOESM2_ESM.docx]

# Additional Analysis for Experiment 1

The relationships between one objective measure (performance accuracy) and two subjective measures (confidence ratings and task ratings) were investigated (see Table 1). A significant positive correlation was identified between confidence ratings and task ratings, *r_s_* = 0.526, *p* < .001. Nevertheless, no significant correlations were identified between accuracy and either confidence ratings or task ratings. This result suggests a dissociation between participants' metacognitive monitoring (both local and global) and their overall objective performance. To further examine the relationship between confidence ratings and objective performance, a more fine-grained analysis was conducted at the trial level. Specifically, we calculated the Goodman and Kruskal’s gamma correlation between confidence ratings (ordinal variable) and response accuracy (binary variable) across all trials and participants. The analysis revealed a significant positive association, γ(5600)=0.349, p < 0.01, indicating that participants tended to report higher confidence when their responses were correct.

This trial-level finding offers an explanation for the previously observed absence of a significant correlation between mean confidence ratings and mean accuracy at the aggregate level. One possible reason is the 50% chance level inherent in the task, which may have led some participants to rely on random guessing, thereby diluting the overall correlation. Additionally, individual differences in the criteria used to assign confidence ratings may have introduced further variability. Such heterogeneity may mask the underlying association at the trial level when data are averaged across individuals, ultimately resulting in a non-significant correlation at the group level.

Table 1

*Descriptive Statistics and Correlations of the Variables from Experiment 1*

|  | *M* (*SD*) | Confidence Rating | Task Rating | Actual Acc. |
| --- | --- | --- | --- | --- |
| Confidence Rating | 3.77 (0.52) | 1 |  |  |
| Task Rating | 6.23 (1.23) | 0.526^**^ | 1 |  |
| Actual Acc. | 0.79 (0.06) | 0.070 | -0.074 | 1 |

**p* < .05, ***p* < .01, ****p* < .001

Participants completed 160 trials totally, with an average number of correct responses of 126.77 (*SD* = 9.55), including 67.89 correct responses (*SD* = 5.26) in the easy condition and 58.89 correct responses (*SD* = 5.70) in the difficult condition. Paired-sample t-tests indicated that the number of correct trials was significantly greater than that of incorrect trials across all conditions (overall: *t*(34) = 28.968, *p* < .001, Conhen’s *d* = 4.897; easy: *t*(34) = 31.383, *p* < .001, Conhen’s *d* = 5.305; difficult: *t*(34) = 19.629, *p* < .001, Conhen’s *d* = 3.318)

To further examine individual task evaluation in local metacognitive monitoring, separate analyses were conducted for correct and incorrect trials (see Table 2). One-sample t-tests revealed that, in correct trials, participants exhibited significantly negative local monitoring biases, indicating a tendency toward underconfidence (overall: *t*(34) = -23.120, *p* < .001, Conhen’s *d* = -3.908; easy: *t*(34) = -20.889, *p* < .001, Conhen’s *d* = -3.531; difficult: *t*(34) = -23.514, *p* < .001, Conhen’s *d* = -3.974). In contrast, local monitoring bias in incorrect trials was significantly positive, suggesting overconfidence in these instances (overall: *t*(34) = 17.981, *p* < .001, Conhen’s *d* = 3.039; easy: *t*(34) = 17.101, *p* < .001, Conhen’s *d* = 2.891; difficult: *t*(34) = 17.111, *p* < .001, Conhen’s *d* = 2.892). Furthermore, paired-sample t-tests indicated no significant differences in the absolute values of local monitoring bias between correct and incorrect trials (overall: *t*(34) = 0.097, *p* = .923, Conhen’s *d* = 0.016; easy: *t*(34) = 1.339, *p* = .190, Conhen’s *d* = 0.226; difficult: *t*(34) = -1.074, *p* = .290, Conhen’s *d* = -0.182). The results showed that bias for correct trials tended to be negative, whereas bias for incorrect trials tended to be positive. However, the difference in their absolute values did not reach statistical significance. Therefore, the overall direction of the monitoring bias may be influenced by the relative proportion of correct (or incorrect) trials within the total number of responses.

Table 2

*Descriptive Statistics of correct and incorrect trial in Local monitoring bias M* (*SD*)

|  | *Total N* | *Local Monitoring Bias* |
| --- | --- | --- |
| Correct trial |  |  |
| Easy | 67.89 (5.26) | -1.88 (0.53) |
| Difficult | 58.89 (5.70) | -2.23 (0.57) |
| Overall | 126.77(9.55) | -2.01 (0.53) |
| Incorrect trial |  |  |
| Easy | 12.11 (5.26) | 2.14 (0.74) |
| Difficult | 21.11(5.69) | 2.07 (0.72) |
| Overall | 33.22(9.55) | 2.10 (0.69) |

*Note.* The "Total N" column indicates the total number of correct and incorrect responses under each sub-condition, whereas the "Local Monitoring Bias" column reflects the values of local metacognitive monitoring bias.

# Additional Analysis for Experiment 2

Participants completed a total of 160 trials. For the high self-esteem group, the average number of correct responses was 127.87 (*SD* = 10.55), with 68.19 correct responses (*SD* = 5.71) in the easy condition and 59.68 correct responses (*SD* = 6.24) in the difficult condition. For the low self-esteem group, the average number of correct responses was 126.87 (*SD* = 8.82), with 68.65 correct responses (*SD* = 4.69) in the easy condition and 58.23 correct responses (*SD* = 5.84) in the difficult condition. Independent-samples t-tests revealed no significant differences between high and low self-esteem groups in the number of correct trials (easy: *t*(60) = 0.340, *p* = .735, Conhen’s *d* = 0.086 , difficult: *t*(60) = -0.946, *p* = .348, Conhen’s *d* = -0.240). Similarly, no significant differences were found between the two groups in the number of incorrect trials (easy: *t*(60) = -0.340, *p* = .735, Conhen’s *d* = -0.086 , difficult: *t*(60) = 0.946, *p* = .348, Conhen’s *d* = 0.240).

Table 3

*Descriptive Statistics of correct and incorrect trial in Local monitoring bias M* (*SD*)

|  |  |  | *Total N* | *Local Monitoring Bias* |
| --- | --- | --- | --- | --- |
| High self-esteem | |  |  |  |
|  | Correct trial | Easy | 68.19 (5.71) | -1.59 (0.53) |
|  |  | Difficult | 59.68 (6.24) | -1.89 (0.54) |
|  |  | Overall | 127.87(10.55) | -1.74 (0.52) |
|  |  |  |  |  |
|  | Incorrect trial | Easy | 11.81 (5.71) | 2.57 (0.73) |
|  |  | Difficult | 20.32(6.24) | 2.56 (0.77) |
|  |  | Overall | 32.12(10.55) | 2.57 (0.70) |
| Low self-esteem | |  |  |  |
|  | Correct trial | Easy | 68.65 (.4.69) | -1.98 (0.66) |
|  |  | Difficult | 58.23 (5.84) | -2.29 (0.66) |
|  |  | Overall | 126.87(8.82) | -2.13 (0.64) |
|  |  |  |  |  |
|  | Incorrect trial | Easy | 11.35 (4.69) | 2.16 (0.75) |
|  |  | Difficult | 21.77(5.84) | 2.23 (0.71) |
|  |  | Overall | 33.13(8.82) | 2.20 (0.70) |

*Note.* The "Total N" column indicates the total number of correct and incorrect responses under each sub-condition, whereas the "Local Monitoring Bias" column reflects the values of local metacognitive monitoring bias.

Additionally, metacognitive efficiency would be calculated, which represents the extent to which individuals are able to discriminate between their correct and incorrect judgements (Maniscalco & Lau, 2014). It is calculated as the ratio of meta-*d*' to *d*', with meta-*d*' reflecting an individual's ability to predict task performance based on confidence ratings, while *d*' representing perceptual sensitivity. The calculation of meta-*d*' typically employs signal detection theory in conjunction with Bayesian inference or maximum likelihood estimation, with the objective of evaluating the relationship between confidence ratings and behavioral performance. The ratio of meta-*d*' to *d*' provides a measure of metacognitive efficiency (M-ratio), which quantifies the efficiency of metacognitive sensitivity in relation to actual task performance. In an ideal scenario, when meta-d' equals d', the M-ratio would be 1, indicating that metacognitive efficiency has reached its optimal level.

Metacognitive efficiency was calculated for both groups to assess whether the pronounced underestimation bias in low self-esteem individuals stemmed from a weaker ability to distinguish between correct and incorrect judgments. It was conducted using metadpy packages developed in Python, which employs maximum likelihood (MEL) estimation and integrates commands from Numpy, Scipy, and Pandas (https://embodied-computation-group.github.io/metadpy/#). The results of the independent samples t-test indicated that there was no statistically significant difference in metacognitive efficiency between the two groups, *t*(60) = 0.512, *p* = .611, Cohen’s *d* = 0.130. The results indicate that the capacity to differentiate between correct and incorrect judgments is not markedly disparate between individuals with high and low self-esteem.

Table 4

*Descriptive statistics of Type 1 sensitivity, Type 2 sensitivity and metacognitive efficiency (M±SD)*

|  | *d’* | meta-*d’* | meta-*d’*/*d’* |
| --- | --- | --- | --- |
| Low self-esteem | 1.72 ± 0.37 | 1.06 ± 0.53 | 0.61 ± 0.29 |
| High self-esteem | 1.78 ± 0.46 | 1.05 ± 0.57 | 0.58 ± 0.28 |

# Additional Analysis for Experiment 3

Participants completed a total of 96 trials in the main phase of the positive and negative conditions, and an average of 64 trials in the main phase of the objective condition. The high self-esteem group achieved an average of 75.22 (*SD* = 7.26), 50.14 (*SD* = 3.78), and 77.19 (*SD* = 6.17) correct trials in the positive, objective, and negative conditions, respectively. The corresponding averages for the low self-esteem group were 74.53 (*SD* = 7.45), 49.64 (*SD* = 4.63), and 74.92 (*SD* = 7.38). Independent-samples t-tests revealed no significant differences between high and low self-esteem groups in the number of correct trials across three feedback conditions (positve: *t*(70) = -0.401, *p* = .690, Conhen’s *d* = -0.094; objective: *t*(70) = -0.502, *p* = .617, Conhen’s *d* = -0.118; negative: *t*(70) = -1.420, *p* = .160, Conhen’s *d* = -0.331).

Table 5

*Descriptive Statistics of correct and incorrect trial in Local monitoring bias M* (*SD*)

|  |  |  | *Total N* | *Local Monitoring Bias* |
| --- | --- | --- | --- | --- |
|  |  |  |  |  |
| High self-esteem | |  |  |  |
|  | Correct trial | Positive | 75.22 (7.26) | -1.44 (0.50) |
|  |  | Objective | 50.14 (3.78) | -1.46 (0.55) |
|  |  | Negative | 77.19 (6.17) | -1.39 (0.57) |
|  |  |  |  |  |
|  | Incorrect trial | Positive | 20.78 (7.26) | 2.94 (0.75) |
|  |  | Objective | 13.86 (3.78) | 3.00 (0.76) |
|  |  | Negative | 18.81 (6.17) | 3.08 (0.76) |
| Low self-esteem | |  |  |  |
|  | Correct trial | Positive | 74.53 (.7.45) | -1.78 (0.66) |
|  |  | Objective | 49.64 (4.63) | -1.91 (0.68) |
|  |  | Negative | 74.92 (7.38) | -2.10 (0.86) |
|  |  |  |  |  |
|  | Incorrect trial | Positive | 21.47 (7.45) | 2.88 (0.73) |
|  |  | Objective | 14.36 (4.63) | 2.69 (0.77) |
|  |  | Negative | 21.08 (7.38) | 2.55 (0.95) |

# Reference

Maniscalco, B., & Lau, H. (2014). Signal Detection Theory Analysis of Type 1 and Type 2 Data: Meta-d′, Response-Specific Meta-d′, and the Unequal Variance SDT Model. In S. M. Fleming & C. D. Frith (Eds.), *The Cognitive Neuroscience of Metacognition* (pp. 25–66). Springer Berlin Heidelberg. https://doi.org/10.1007/978-3-642-45190-4_3
